# Evidence for the Involvement of the Master Transcription Factor NF-κB in Cancer Initiation and Progression

**DOI:** 10.3390/biomedicines6030082

**Published:** 2018-07-27

**Authors:** Yu Rou Puar, Muthu K Shanmugam, Lu Fan, Frank Arfuso, Gautam Sethi, Vinay Tergaonkar

**Affiliations:** 1 Department of Pharmacology, Yong Loo Lin School of Medicine, National University of Singapore, Singapore 117600, Singapore; yu.rou.puar@gmail.com (Y.R.P.); phcsmk@nus.edu.sg (M.K.S.); phcfanl@nus.edu.sg (L.F.); 2 Stem Cell and Cancer Biology Laboratory, School of Biomedical Sciences, Curtin Health Innovation Research Institute, Curtin University, Perth, WA 6009, Australia; frank.arfuso@curtin.edu.au; 3 Institute of Molecular and Cellular Biology (A*STAR), 61 Biopolis Drive, Singapore 138673, Singapore; 4 Department of Biochemistry, Yong Loo Lin School of Medicine, National University of Singapore, Singapore 117597, Singapore; 5 Centre for Cancer Biology (University of South Australia and SA Pathology), Adelaide, SA 5000, Australia

**Keywords:** NF-κB, cancer, apoptosis, metastasis, pharmacological inhibition

## Abstract

Nuclear factor kappa-light-chain-enhancer of activated B cells (NF-κB) is responsible for the regulation of a large number of genes that are involved in important physiological processes, including survival, inflammation, and immune responses. At the same time, this transcription factor can control the expression of a plethora of genes that promote tumor cell proliferation, survival, metastasis, inflammation, invasion, and angiogenesis. The aberrant activation of this transcription factor has been observed in several types of cancer and is known to contribute to aggressive tumor growth and resistance to therapeutic treatment. Although NF-κB has been identified to be a major contributor to cancer initiation and development, there is evidence revealing its role in tumor suppression. This review briefly highlights the major mechanisms of NF-κB activation, the role of NF-κB in tumor promotion and suppression, as well as a few important pharmacological strategies that have been developed to modulate NF-κB function.

## 1. Introduction

The nuclear factor kappa-light-chain-enhancer of activated B cells (NF-κB) is a family of transcription factors that was first discovered by David Baltimore in 1986 and has recently generated considerable interest for its role in the development of a variety of human ailments. In mammalian cells, it consists of five members, namely, RelA (p65), RelB, Rel (c-Rel), NF-κB1 (p50/p105), and NF-κB2 (p52/p100) that can form both homodimers and heterodimers and play an essential role in the regulation of immune responses and inflammation [1,2,3,4]. In non-stimulated cells, NF-κB is associated with IκB proteins and resides in the cytoplasm [5,6,7].

## 2. Activation Pathways

There are various modes of NF-κB activation that have been documented in the literature [8]. The activation of NF-κB through the canonical pathway, the most widely known NF-κB pathway, is essential for inflammation and innate immunity [9,10,11]. This involves the phosphorylation of the IκB protein on two conserved serine residues within its N-terminal domain. This is carried out by two IκB kinases, IKK1 and IKK2, which reside in the IKK complex. The IKK complex varies in size and composition in different cell types and can also contain non-enzymatic regulatory subunit IKKy (NEMO), which is essential for the activation of the IKK1-IKK2 heterodimer [12]. The phosphorylation of these residues leads to rapid polyubiquitination by the Skp, Cullin, F-box β-transducin repeat-containing protein (SCF-β-TrCP) complex and subsequent degradation by the 26S proteasome, activating the NF-κB signaling cascade and resulting in the complete degradation of IκB proteins [13,14,15,16]. The NF-κB dimers then undergo further modification through phosphorylation and acetylation before translocation to the nucleus where they bind to the DNA and recruit transcriptional coactivators [12,17,18] and are thus constitutively activated in various human diseases (Table 1).

**Figure 1:**
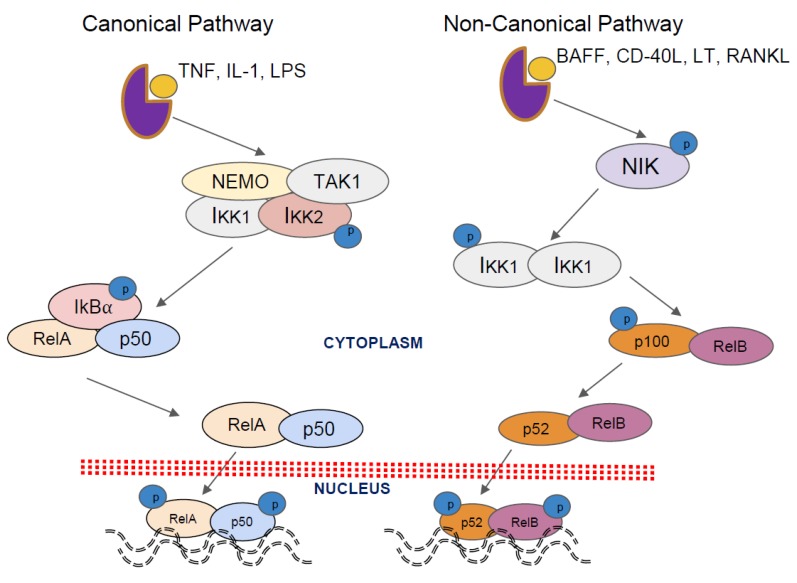
A brief overview of canonical and non-canonical nuclear factor kappa-light-chain-enhancer of activated B cells (NF-κB) activation pathways. Canonical signaling pathway: Upon receptor engagement, IκB kinase 1 [KK1] (α) and IκB kinase 1 and IκB kinase 2 [IKK2] (β) are activated through phosphorylation before proceeding to phosphorylate nuclear factor of kappa light polypeptide gene enhancer in B-cells inhibitor, alpha (IκB) members. Phosphorylation of these residues leads to Skp, Cullin, F-box β-transducin repeat-containing protein SCF-β-TrCP complex-mediated rapid polyubiquitination and subsequent degradation by the 26S proteasome. Non-canonical signaling pathway: upon receptor engagement, NF-κB-inducing kinase (NIK) is activated and directly phosphorylates and activates the IKK1 homodimer, which goes on to phosphorylate p100, leading to the partial SCF-β-TrCP-mediated degradation of p100 to generate the p52-RelB complex.

The activation of NF-κB through the non-canonical pathway is essential in lymphoid organ development and adaptive immunity [19,20], (Figure 1). This pathway involves a more restricted set of ligands, such as the B-cell-activating factor (BAFF), lymphotoxin-beta (LT-β), and cluster of differentiation 40 (CD40), which can lead to the activation of the NF-κB inducing kinase (NIK) that phosphorylates and activates the IKK1 homodimer, resulting in the phosphorylation of p100 nd the subsequent degradation to p52 [2,20,21]. Upon p100 degradation, the RelB/p50 and RelB/p52 dimers are released and translocated into the nucleus where they start the transcription of the target genes [20,22,23].

While the canonical and non-canonical pathways have been the target of the majority of the research done on NF-κB activation, there have been an increasing number of alternative mechanisms, such as IKK-independent processes, that have been shown to activate NF-κB in a manner that is completely different from that of the canonical and non-canonical pathways [2,24,25].

Most carcinogens, including cigarette smoke, alcohol, and UV light, as well as nearly all infectious agents such as human immunodeficiency virus (HIV) and Hepatitis B and C viruses, have been shown to activate NF-κB [26,27,28,29,30,31,32], (Table 2). Interestingly, the epidermal growth factor (EGF) has been previously shown to induce NF-κB activation by inducing tyrosine phosphorylation of nuclear factor of kappa light polypeptide gene enhancer in B-cells inhibitor, alpha (IκB⍺) at residue 42 [33]. While the tumor necrosis factor (TNF)-induced NF-κB activation was found to be IKK dependent, it has been shown that chemotherapeutic agents and radiation may also activate NF-κB through diverse IKK-independent mechanisms [2,11,33].

## 3. NF-κB Activation by Chemotherapeutic Agents

Several chemotherapeutic agents, including paclitaxel, vinblastine, vincristine, doxorubicin, and daunomycin have been reported to induce NF-κB activation in different cells However, it has also been found that the nature of the genes expressed following chemotherapy-induced NF-κB activation is context dependent and this activation can result in both the expression or suppression of anti-apoptotic genes [34,35,36,37].

Topoisomerase poisons found in numerous chemotherapeutic agents (actinomycin D, camptothecin, daunomycin, and etoposide), which have the common property of generating DNA strand breaks, can activate NF-κB in leukemia cells [35,38]. SN38 (7-ethyl-10-hydroxycamptothecin) and doxorubicin have been shown to trigger the activation of NF-κB through a pathway that involves the phosphorylation and degradation of IκBα [39]. Interestingly, adriamycin was also found to activate NF-κB in human small-cell lung carcinoma cells in a non-specific, dose-dependent manner, similarly involving the degradation of IκBβ [40]. Moreover, incubation of multiple myeloma cells with doxorubicin or the alkylating agent melphalan can lead to the robust activation of NF-κB activity that confers anti-apoptotic abilities to the treated cells [41].

There are conflicting reports regarding the role of the different IKK subunits in the NF-κB activation induced by doxorubicin [11,42,43]. On the one hand, it has been shown that TAK1 is required for doxorubicin-induced NF-κB activation, with doxorubicin inducing Lys63-linked TAK1 polyubiquitination at the lysine 158 residue during the initial stages of treatment [44]. Interestingly, at the later stage of doxorubicin exposure, Lys48-linked TAK1 polyubiquitination was observed to induce TAK1 degradation. However, it has also been reported that NF-κB complexes produced through doxorubicin-induced NF-κB activation may contribute to the suppression of constitutive- and cytokine-induced NF-κB dependent transcription. RelA produced through this pathway is not phosphorylated or acetylated and is responsible for blocking NF-κB signaling in a histone deacetylase-independent manner. In addition to this, upon doxorubicin-induced NF-κB activation, this transcription factor may no longer remain stably bound to κB elements in vivo; thus, suggesting that doxorubicin-induced NF-κB activation may not necessarily contribute to the anti-apoptotic activity in cancer cells [45].

Microtubule-disrupting agents have also been found to activate NF-κB in diverse models. Both paclitaxel and vinca alkaloids were found to induce NF-κB activation through the degradation and down-regulation of IκBα [35,46]. Paclitaxel is known to induce TNF and interleukin-1 expression in a manner that is similar to that of lipopolysaccharides, which can induce NF-κB activation via a pathway that involves TNF expression; hence, it has been proposed that paclitaxel-induced NF-κB activation utilizes a similar pathway. However, anthracyclines and vinca alkaloids are similarly able to induce NF-κB activation despite not up-regulating TNF or interleukin-1 gene expression, thus, suggesting that chemotherapy-induced NF-κB activation may not always involve cytokine up-regulation [35]. Thus, chemotherapeutic agents have been reported to modulate NF-κB activation by diverse molecular mechanism(s).

## 4. NF-κB Activation by Radiation

There are numerous studies supporting the involvement of ionizing radiation (IR) in the activation of NF-κB and the development of anti-apoptotic abilities in cancer cells [35,47,48,49,50,51,52,53,54,55,56,57,58,59,60]. IR-induced NF-κB activation has been observed to be initiated by the proteasomal degradation and phosphorylation of IκB by the IKK complex [61].

It has been shown that the IR sensitivity of tissues towards the activation of NF-κB in vivo is context dependent and varies depending on the type of tissue [35]. The IR doses required for maximal NF-κB activation vary greatly depending on the cell lines or systems analyzed, ranging from 0.5 GY to over 20 GY [62,63]. There are contrasting observations of NF-κB activation following whole body irradiation at different IR doses [50,51]. NF-κB activation by IR (8.5 GY) has been shown to be tissue-specific and only detectable in the bone marrow, lymph nodes, and spleen [50]. However, another report demonstrated the activation of NF-κB in the liver and kidney when mice were exposed to 20 Gy of IR [64]. These diverse findings suggest that different normal tissues possess differential IR sensitivities towards the activation of NF-κB.

It has been proposed that a possible mechanism through which IR induces NF-κB activation involves nuclear DNA damage and a pathway similar to the one used by camptothecin, an anti-cancer agent, that causes DNA double-stranded breaks that trigger a signaling cascade that results in the degradation of IκB⍺ and the activation of NF-κB [65]. Interestingly, other studies have indicated that IκBα degradation was not observed in response to ionizing radiation exposure in cells from patients with ataxia-telangiectasia [49,66].

DNA-dependent protein kinase (DNA-PK) has also been shown to be essential for IR-induced NF-κB activation [48]. There are conflicting reports regarding the role of the IκB kinase complex, and the phosphorylation and degradation of IκB by the ubiquitin-proteasome pathway to release active NF-κB when cells are exposed to different doses of IR in differential cell systems [35,64,67,68,69].

While it has been shown that both exposure to short wavelength UV (UV-C) and gamma radiation induces NF-κB activation through a ubiquitin/proteasome pathway, UV-C-induced NF-κB activation was found to involve the degradation of IκB through phosphorylation at Ser-32 and Ser-36, leading to the activation of IKK, whereas gamma rays-induced NF-κB activation was found to utilize a different pathway; thus, suggesting that both types of radiation may utilize two different mechanisms to activate NF-κB [51]. Constitutive nitric oxide synthase activation following IR in a therapeutic dose range has been shown to cause the nitration and dissociation of IκBα tyrosine 181 from NF-κB in a process that does not involve the phosphorylation or degradation of IκBα [70]. 

While the activation of NF-κB has long been associated with anti-apoptotic abilities in cancer cells, NF-κB has been found to be required for cell death following UV stimulation. The exposure of U2OS bone osteosarcoma cells to UV stimulation triggered the activation of NF-κB as well as the induction of p53. However, this UV-induced NF-κB was found to be transcriptionally inert, suggesting that p53 may have a role to play in the switching of NF-κB from its usual anti-apoptotic role to a pro-apoptotic role within the cell, possibly due to its ability to sequester transcriptional co-activator proteins such as p300/CREB [35,71].

The activation of NF-κB by UV-irradiation occurs in two phases; with the early phase peaking with the greatest level of DNA binding observed at 4 h post-irradiation and the late phase occurring between 16 and 48 h post-irradiation [58]. Although IκB⍺ depletion was observed during the late-phase of UV-irradiation, neither the ubiquitination nor the proteasomal cleavages were reported to have detectable attributions to the late-phase IκBα depletion. Instead, it has been suggested that the late phase activation of NF-κB may be regulated through a protein kinase A (PKA)/mitogen- and stress-activated protein kinase (MSK) pathway [35,58]. Overall, the exposure to radiation can regulate NF-κB activation through different modes of actions.

## 5. NF-κB Signaling Pathway in Inflammation

The NF-κB pathway has long been associated with inflammation due to its activation by pro-inflammatory cytokines as well as its involvement in the activation of numerous pro-inflammatory genes [20,23]. Inflammation triggered both by hepatitis and cancer progression has been shown to up-regulate TNFα, a pro-inflammatory cytokine, in Md2-knockout mice. While the inhibition of NF-κB had no effect on the development of transformed hepatocytes in the liver of the mice, the suppression of NF-κB was shown to promote the apoptosis of transformed hepatocytes and prevented the progression to hepatocellular carcinoma, suggesting that NF-κB plays a crucial role in cancer development [32,72]. However, studies have shown that the NF-κB activation is not solely pro-inflammatory and, depending on the context, can also play a part in the anti-inflammatory responses within the cells [15,16,32]. While NF-κB has been shown to initiate pro-inflammatory gene expression when activated during the onset of inflammation in leukocyte cells, interestingly, it has also been shown to be crucial for the activation of anti-inflammatory genes during the resolution of inflammation. When NF-κB activation was inhibited during the resolution of inflammation, it was found to inhibit apoptosis and protract the inflammatory process [73].

## 6. NF-κB as a Tumor Promoter and Suppressor

**Figure 2:**
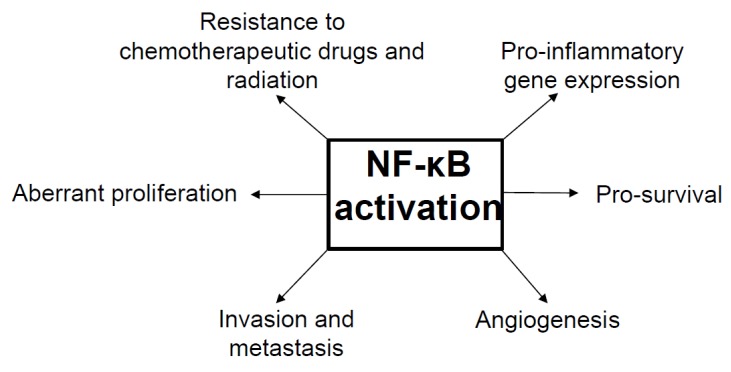
A schematic diagram depicting the potential role of NF-κB in cancer initiation and progression.

It has been shown that the uncontrolled activation of NF-κB contributes to the initiation of tumorigenesis and plays a crucial role in tumor cell proliferation and survival [20,23,74,75,76,77], (Figure 2). NF-κB activation is essential in protecting transformed cells from macrophage-induced apoptosis during tumor initiation through the upregulation of TNF and nitric oxide [78]. In addition to playing a crucial role in tumor initiation, NF-κB has also been found to play a role in allowing cancer cells to avoid detection by the adaptive immune cells. The prolonged activation of NF-κB promotes tumorigenic proliferation and metastasis through inducing the expression of proto-oncogenes, such as *c-myc* and *cyclin D1*, as well as cell adhesion molecules, vascular endothelial growth factors (VEGFs), and matrix metalloproteinases (MMPs) [15,16]. It has been shown that the inhibition of NF-κB abolishes VEGF production and angiogenesis in a variety of conditions. Furthermore, the basic fibroblast growth factor (bFGF), interleukin-8 (IL-8), MMP-9, and other NF-κB target genes are involved in multiple steps of angiogenesis [15,16,79,80]. It is worth noting that MMPs including MMP-2, -3, and -9 degrade the basement membrane and remodel the extracellular matrix, which facilitates cell migration and favors either angiogenesis (endothelial cells) or metastasis (cancer cells) in different microenvironment [80,81]. Interestingly, human telomerase reverse transcriptase, the catalytic subunit of telomerase [82,83,84,85] was found to drive gastric intestinal metaplasia by upregulating CDX2 (caudal type homeobox 2) expression via NF-κB signaling axis [86].

Morgana, a *CHORDC1* gene product and a component of the IKK complex, has been shown to play an integral role in NF-κB activation and tumorigenesis. High levels of Morgana have been shown to promote tumor metastasis, induce the expression of cytokines, and suppress the presence of natural killer cells during the initial tumor growth stage as well as during the pre-metastatic stage in breast cancer mouse models, thereby promoting tumor growth and cancer progression [87]. NF-κB has also been demonstrated to play a part in the upregulation of the expression of chemokine receptor type 4 (CXCR4), a stromal cell-derived factor 1 alpha receptor, in highly metastatic breast cancer cells that contribute towards tumor growth [88,89].

The p65 and p50 NF-κB subunits were found to bind directly to the CXCR4 promoter and initiate transcription, and increased CXCR4 cell surface expression was also associated with cancer cell metastasis [88,89,90,91]. Epithelial-mesenchymal transition (EMT) is an early event in metastasis [80,92,93,94]. TNF-α in the tumor microenvironment acts as an inflammatory mediator that triggers the EMT of tumor cells and promotes tumor metastasis. In oral cancer cells, it promotes cell invasion and metastasis, which rely on the NF-κB signaling pathway activation [95,96,97,98]. Cell adhesion molecules such as selectins, integrins, and their ligands can also be regulated by the NF-κB pathway [80,99], and are important in promoting cancer cell extravasation and colonization at distant sites, although the mechanistic details remain elusive [100].

However, in several specific cases, NF-κB may also function as a potential tumor suppressor. p65 has been shown to be capable of switching from its role as a tumor suppressor to a tumor promoter depending on the progression of tumorigenesis, with the regulation occurring in a cell autologous manner [78]. Interestingly, it was noted that targeted knockout of IKK2 in hepatocytes can promote the carcinogenesis in the diethylnitrosamine-induced hepatocellular carcinoma mouse HCC model [101]. Additionally, NEMO deletion was found to induce hepatitis, fibrosis, and liver tumorigenesis [102]. The elimination of NF-κB activity in hepatocytes was shown to promote inflammatory cytokine expression and increase tumor formation in animals tested, indicating the vital role that NF-κB plays in suppressing tumor formation and growth [102]. Interestingly, the activation of IKKα has been found to mediate tumor suppression in human squamous cell carcinomas of the skin, lungs, and head and neck [15,16,103,104,105,106].

c-Jun N-terminal kinase (JNK) is a kinase that is responsible for the phosphorylation of proteins involved in both apoptotic and anti-apoptotic activity in cancer cells. The prolonged activation of JNK gives rise to the characteristic uncontrolled proliferation often observed in tumor cells [107,108]. Studies have shown that transient transfection of the kinase-mutated IKKβ into human bronchial epithelial cells resulted in enhanced JNK activation following IKKβ-NF-κB inhibition. Reactive oxygen species (ROS) have been suggested to play an important role in TNFα or arsenic-induced JNK activation in cells, during which the NF-κB pathway may be inhibited. NF-κB activation has therefore been suggested to be crucial in preventing cells from suffering from oxidative stress through curbing ROS generation and thereby preventing JNK activation [109,110].

## 7. Inhibitors of NF-κB Function and Selected Pharmacological Strategies to Block NF-κB Function

A plethora of compounds consisting of small molecules, biologics, inhibitory peptides, and many other different types of bioactive molecules have been identified as inhibitors of NF-κB and categorized into different groups based on the stage of NF-κB activation at which they exert their inhibitory effects [111,112,113,114]. These groups include agents that act at various steps of NF-κB signaling at (i) upstream of IKK, (ii) directly affecting the IKK complex or IκB phosphorylation, (iii) ubiquitination or proteasomal degradation of IκB, (iv) nuclear translocation of NF-κB, (v) NF-κB DNA binding, and (vi) NF-κB-directed gene transactivation.

### 7.1. Inhibitors That Act Upstream of the IKK Complex

Since the IKK complex is usually involved in the initial stages of the pathways leading to NF-κB activation, one viable strategy for inhibition NF-κB activation would be to block a signal upstream of IKK to prevent it from activating the IKK complex [2,81,114].

TNF-Rs comprise a family of 29 structurally-related receptors, which are bound by 19 ligands of the TNF superfamily [115,116,117,118,119]. The usage of anti-TNF antibodies or agents that block the TNF-R, such as infliximab and etanercept, can inhibit TNF-induced NF-κB activation and can have benefits in various autoimmune diseases [115,120,121]. Infliximab, a chimeric anti-TNF⍺ antibody, is capable of inhibiting TNF-induced inflammation through binding to membrane TNF and preventing TNF binding to its receptors [115,122,123]. Etanercept, a TNF-receptor: lg fusion protein is similarly capable of blocking the TNF’s cytotoxicity and ability to induce inflammation through binding to human TNF [115]. However, patients treated with these drugs often experience significant side effects, including fevers, hypotension, and nausea [115,124,125,126]. The risks associated with the use of anti-TNF antibodies include the possibility of the development of anti-drug antibodies that can result in the loss of clinical response as well as other adverse drug reactions, including acute hypersensitivity [127,128,129]. However, it has been found that co-administration of immune suppressants, such as methotrexate, generally reduces the incidence of anti-drug antibodies [130]. The careful monitoring of disease progression, along with anti-drug antibody monitoring is therefore crucial in ensuring the safe use of anti-TNF antibodies [131].

Bruton’s tyrosine kinase (BTK) protein is essential for B proliferation in response to B cell antigen receptor (BCR) stimulation. BCR exposure can lead to the activation of NF-κB which, in turn, can regulate various genes controlling B cell growth. In both transformed and primary B cells, the absence of BTK severely limited B cell antigen receptor (BCR)-induced NF-κB activation. The loss of BTK in cells has also been associated with defects in the nuclear translocation of RelA and c-Rel, both crucial transactivating subunits of NF-κB in B cells [132,133,134]. For example, Ibrutinib (PCI-32765) binds to the cysteine residue 481 in the BTK active site, thereby inhibiting BTK phosphorylation on tyrosine 223 and preventing BTK activity [135].

While cases of primary and secondary resistance towards ibrutinib in B-cell malignancies have emerged, and mutations within BTK have been observed to affect the efficacy of the drug, there have been alternative mechanisms identified that provide the option to bypass BTK entirely, thus providing alternative options for other targeted agents [136]. The gene encoding the adaptor protein MYD88, which is responsible for the activation of toll-like receptors (TLRs), is frequently observed to be mutated in hematological malignancies where it can induce constitutive NF-κB activation, thus making TLR signaling a viable target for therapeutic efficacy [137,138].

IMO-8400, an antisense oligonucleotide TLR inhibitor, has been shown to specifically target TLR7, TLR8, and TLR in preclinical studies and is effective in reducing the size of Waldenstrom macroglobulinemia (WM) and diffuse large B-cell lymphoma (DLBCL) xenografts, driven by gain-of-function MYD88 mutations [138]. A phase I/II trial of IMO-8400 is ongoing in patients in WM and DLBCL, and second generation TLR 7/TLR 8/TLR9 inhibitors are currently in development. Interestingly, IMO-8400 was also found to exhibit clinical efficacy in a phase 2a, randomized, placebo-controlled trial in patients with moderate-to-severe plaque psoriasis [139].

### 7.2. IKK Inhibitors

As the IKK complex plays a crucial role in signal integration for NF-κB activation pathways, it has attracted much interest and research into compounds that are able to block IκB phosphorylation and, hence, also prevent ubiquitination of IκBα and its further degradation [25,140,141]. However, while many inhibitors have been developed, few have managed to enter into clinical trials and none have been clinically approved [141,142]. However, it must also be noted that while the targeting IKK2 holds promise as a likely anti-inflammatory therapy, it was found that pharmacological IKK2 inhibition can also result in increased endotoxin susceptibility that is associated with increased levels of IL-1β as a result of increased pro-IL-1β secretion by macrophages and neutrophils upon bacterial infections, thereby causing overt systemic inflammation and lethality in mice [143].

Although numerous agents have been identified to be involved in the inhibition of NF-κB activation at the IKK step, the exact mechanism through which all these agents inhibit IKK is still not fully understood [25,141]. Of the few IKKα/β inhibitors that have been investigated, the mechanisms of action identified can be classified into three categories: adenosine triphosphate (ATP) analogues, which show some specificity for interacting with IKK; compounds that have allosteric effects on IKK structure; and agents interfering with the kinase activation loops [25,144,145,146].

ATP analogs include natural products such as β-carboline and synthetic compounds such as SC-839, which has an approximately 200-fold preference for IKKβ compared to IKKα [147,148]. BMS-345541 is a synthetic compound capable of exerting an allosteric effect on the IKK structure and has been observed to inhibit ATP binding to IKKα as well as inhibiting the expression of cytokines including TNFα, IL-1β, -8, and -6 [25,145,146,149,150]. In addition, various natural products including curcumin, pinitol, honokiol, mangiferin, etc. have also been reported to modulate IKK activation in diverse tumor cell lines [141].

The interaction between the C-terminus NEMO binding domain of the IKK complex and NEMO is a crucial step in the activation of the IKK complex, hence, it is an attractive target for the prevention of IKK complex formation and subsequent NF-κB activation. Phenothiazine 22 and its related analogs (22.2, 22.4, and 22.10) have been identified as potential drugs to be used to inhibit NF-κB activation due to their ability to reduce nitrite production and inducible nitric oxide synthase mRNA expression when administered to murine macrophages stimulated with lipopolysaccharide. These effects have been observed to be accompanied by NF-κB inhibition as well as the decreased expression of phosphorylated IKKβ, IκBα, and NF-κB/p65 [151].

Dominant-negative forms of IKKα and IKKβ are also able to function as inhibitors to modulate IKK activation as they are capable of showing stimulus-dependent inhibition due to their role in both the canonical and non-canonical pathways [25,145,152,153,154].

### 7.3. Proteasomal Degradation of IκBα

Since the ubiquitination of IκB by the SCF-B-TrCP ubiquitin ligase complex and the subsequent degradation by the 26S proteasome is a crucial step in the NF-κB activation pathways, preventing the degradation of IκBα is a potential method of treatment to prevent NF-κB activation [13,14,141,155,156].

Among IκB ubiquitination blockers, the virulence factor YopJ can act as a promiscuous deubiquitinating enzyme and is able to negatively regulate signaling by removing ubiquitin moieties from critical proteins such as the TNF receptor-associated factor (TRAF)2, TRAF6, and IκBα [157].

Peptide boronic acids named PS-262, PS-273, PS-341, and PS-402 were originally used as inhibitors of serine proteases but were noted to act as proteasome inhibitors by blocking the chymotrypsin-like site in the 20S subunit core and to be more potent than their aldehyde analogs [158,159,160]. Bortezomib is one such inhibitor that has been shown to prevent tumor growth and promote cell apoptosis in numerous cancers [141,161,162,163,164]. Carfilzomib, a tetrapeptide ketoepoxide, has also been demonstrated to induce apoptosis in chronic lymphocytic leukemia patient cells in the presence of human serum [165]. However, reports have revealed that bortezomib is also capable of downregulating IκBα expression and promoting NF-κB activation via the canonical pathway in multiple myeloma cell lines and primary tumor cells from patients [166]. Similarly, proteasome inhibitors MG-115, MG-132, and lactacystin were also shown to promote NF-κB activation in cells through an increase in IKK activation and IκBα degradation, further highlighting how the use of proteasome inhibitors to prevent NF-κB may not necessarily block NF-κB activation in cancer cells [141,167].

### 7.4. NF-κB DNA Binding

One method for inhibiting NF-κB activation through the prevention of NF-κB DNA binding involves the use of small peptides that are able to permeate the cell membrane to block NF-κB nuclear translocation [2,81,114,168,169]. SN-50, a forty-one-residue synthetic peptide containing a hydrophobic membrane-translocating region and the nuclear localization sequence of NF-κB p50, has been shown to inhibit NF-κB activation at high concentrations through the saturation of the transport machinery responsible for importing p50-containing dimers into the nucleus [145,146,170]. However, despite its ability to inhibit NF-κB activation, the high peptide concentration required to achieve the desired inhibition, along with the non-specific nature of the inhibition during which other unrelated transcription factors are similarly affected, limits the ability for the peptide inhibitor to be used in treatment [2,81,114,171]. A cell-permeable peptide containing the NF-κB nuclear localization sequence (NLS) was found to specifically block the importin α-mediated nuclear import of NF-κB, thereby reducing the effects of inflammation in vascular smooth muscle cells and macrophages. Plaques from NLS-treated mice were also found to contain fewer macrophages of the pro-inflammatory M1 subtype than those from respective untreated controls, thereby indicating the potential of NLS to target NF-κB nuclear translocation and prevent inflammation [172].

Numerous sesquiterpene lactones (SLs) have been found to contribute to the inhibition of NF-κB by preventing inflammation and blocking RelA-containing NF-κB dimers from binding to DNA by interacting with C38 within RelA’s DNA-binding loop 1 [145,146]. While it has been suggested that SLs exert their inhibitory effect through the degradation of IκB, when okadaic acid-stimulated cells were treated with helenalin, no IκB degradation or NF-κB nuclear translocation was observed and this SL was found to selectively alkylate the p65 subunit of NF-κB, suggesting that SLs may possibly exert their effects through directly modifying NF-κB [173].

Decoy oligodeoxynucleotides (ODNs) are also capable of preventing NF-κB DNA binding through binding to specific genomic promoters with their κB sites, thereby preventing NF-κB dimer binding [2,81,114,174,175,176]. Treatment using ODNs has been reported to be successful in numerous animal models of inflammation and cancer, and can be pursued further in clinical settings [2,81,114,146,177,178,179].

### 7.5. Non-Steroidal Anti-Inflammatory Drugs and Antioxidants

Non-steroidal anti-inflammatory drugs (NSAIDs) such as aspirin have been shown to suppress NF-κB, which controls the expression of genes such as cyclooxygenase (COX)-2 and cyclin D1, leading to the inhibition of proliferation of tumor cells [114,180]. Interestingly, NSAIDs have been found to differ in their ability to suppress NF-κB activation, with aspirin and ibuprofen being the least potent and with resveratrol, curcumin, celecoxib, and tamoxifen being the most potent amongst the agents investigated [181]. There is also compelling evidence to suggest that c-Src may be an upstream mediator of aspirin/NSAID effects on NF-κB signaling and apoptosis in colorectal cancer cells [182]. However, it was also found that hepatocytes are not sensitive to NF-κB inhibition by NSAIDs and that these drugs, especially the COX-2 selective inhibitors, do not survival [183]. In another study, it was reported that a new H_2_S-releasing derivative of naproxen, ATB-346 [2-(6-methoxynapthalen-2-yl)-propionic acid 4-thiocarbamoyl phenyl ester] can abrogate proliferation and induce apoptosis via the negative regulation of NF-κB activation in human melanoma cells [184]. Moreover, the co-administration of three different NSAIDs (celecoxib, etoricoxib, and diclofenac) was found to significantly abrogate the development of the 1,2-dimethylhydrazine dihydrochloride-induced colorectal cancer via the suppression of the NF-κB activation cascade [185].

### 7.6. Gene Therapy Approaches

Gene therapy involves the use of therapeutic vectors to target NF-κB via a long-term approach and has been heavily researched as a potential treatment for cancer. These therapeutic vectors can be administrated either systemically or locally at the site of inflammation, though the latter approach is deemed as a safer one due to the reduced risks of toxic side-effects as well as its ability to maintain constant therapeutic levels in the target tissue [77,186,187].

Viral-mediated gene transfer is an effective method for administering therapeutic proteins in vivo, with the type of vector being chosen based on cell type and nature of the disease in order to maximize the therapeutic effects [188,189,190]. Adenoviral vectors used in the intra-articular gene transfer of a dominant-negative adenoviral IKKβ construct (Ad.IKKBdn) was shown to significantly ameliorate the severity of adjuvant arthritis in mice and was accompanied by a significant decrease in NF-κB DNA expression in the joints of the treated animals [191]. The adenovirus vector expressing the dominant negative mutant of IKKβ was also found to be a viable anti-cancer therapy and sensitized human prostate carcinoma cells, neuroblastoma cells, and lung cancer cells to TRAIL- or TNF-induced apoptosis, suggesting that targeting NF-κB at the level of IKKβ through the use of adenoviral vectors appears promising [192,193,194].

Recombinant adenoviruses (Ads) are effective vectors for gene transfer due to their ability to infect numerous different types of tissues and cell types without needing a replicating target cell. Most recombinant Ads have deletions of early region 3 (E3) genes, allowing more space for insertion of the transgene. The E3 region of Ads has been shown to inhibit the activation of NF-κB induced by TNF-α and IL-1, as well as preventing NF-κB from entering the nucleus and preventing the activation of IKK [195].

Decoy ODNs are short synthetic fragments of DNA or RNA, mimicking complementary sequences of nucleic acids or transcription factors, and thus preventing the transcription factors from binding to the target gene promoter region. They have been shown to suppress NF-κB activation as well as mRNA expression of TNF-α, IFN-y, and ICAM-1 in liver grafts, with a significantly lower hepatic NF-κB DNA binding activity [196]. However, the therapeutic use of ODNs is strongly hampered by their low bioavailability and short half-life [77,186,187,197]. While different strategies involving chemical modifications of the nucleic acid backbone and use of delivery systems have been investigated to overcome the pharmacokinetic drawbacks of ODNs, many chemical modifications have met with unsuccessful results [186,198].

RNA interference (RNAi) is the specific suppression of genes by short, double-stranded RNA. Small interfering RNA (siRNA) has been shown to be capable of inhibiting NF-κB activation through decreasing p65 and IKK1-IKK2 expression [199,200]. The transfection of siRNA targeting p65 was also shown to significantly inhibit NF-κB activation, induce cell cycle arrest, cell death, and sensitize head and neck squamous cell carcinoma cells when combined with histone deacetylase inhibitors [201]. However, it must also be noted that vector-based, sustained high-level delivery of siRNAs can lead to dose-dependent liver injury and even death in mouse models [202].

## 8. Conclusions

NF-κB has gained much recognition for its role in cancer and as one of the most important pro-inflammatory transcription factors. Although extensive research successfully supports and demonstrates the efficacy of NF-κB inhibitors in adjuvant therapy, the potential undesirable side-effects of prolonged NF-κB inhibition must also be taken into account when proposing novel treatment plans. NF-κB inhibitors should also always be tested and used with caution due to the context-dependent nature of NF-κB function in cells along with its tumor promoting and pro-survival abilities. While NSAIDs, corticosteroids, and many other drugs are currently being used for treating inflammatory conditions and cancers, it should be noted that these drugs often lack specificity for inhibiting NF-κB activity and consequently require relatively high concentrations. Thus, the identification of novel approaches to target this master transcription factor is still needed.

## Figures and Tables

**Table 1:** The activation of nuclear factor kappa-light-chain-enhancer of activated B cells (NF-κB) in selected human diseases.

Cancers	Inflammatory Diseases
Acute lymphoblastic leukemia Anaplastic large-cell lymphoma Breast Burkitt lymphoma Cervical Colorectal Diffuse large B-cell lymphoma Fibrosarcoma Head and neck Hodgkin’s lymphoma Mammary carcinoma Mantle cell lymphoma Melanoma Multiple myeloma Lung Ovarian Pancreatic Prostate Squamous-cell carcinoma Thyroid Vulva	Alzheimer’s disease Rheumatoid arthritis Atherosclerosis Multiple sclerosis Chronic inflammatory demyelinating Polyradiculoneuritis Asthma Inflammatory bowel disease Helicobacter pylori-associated gastritis Systemic inflammatory response syndrome Parkinson’s disease

**Table 2:** A list of a few important stimuli activating NF-κB.

Class	Inducing Stimuli
Viruses	Human immunodeficiency virus Hepatitis B virus Human herpes virus 6 Adenovirus
Viral products	Double-stranded RNA Latent membrane protein Hepatitis B viral protein HBx Middle hepatitis Bvirussurfaceprotein MHBs
Inflammatory cytokines	Tumor necrosis factor-α Lymphotoxin Interleukin-1 Interleukin-2 Leukotriene B4
Bacterial products	Lipopolysaccharide Exotoxin B Toxic shock syndrome toxin 1 Muramyl peptides
Physical stress	UV light
